# Examining how p16^*INK4a*^ expression levels are linked to handgrip strength in the elderly

**DOI:** 10.1038/srep31905

**Published:** 2016-08-23

**Authors:** Tung-Wei Kao, Wei-Liang Chen, Der- Sheng Han, Ying-Hsin Huang, Chi-Ling Chen, Wei-Shiung Yang

**Affiliations:** 1 Division of Geriatric Medicine, Department of Family and Community Medicine, Tri-Service General Hospital, National Defense Medical Center, Taipei, Taiwan; 2 Graduate Institute of Clinical Medicine, College of Medicine, National Taiwan University, Taipei, Taiwan; 3 Department of Physical Medicine and Rehabilitation, National Taiwan University Hospital BeiHu Branch, Taipei, Taiwan; 4 Department of Internal Medicine, National Taiwan University Hospital, Taipei, Taiwan; 5 Research Centre for Developmental Biology and Regenerative Medicine, National Taiwan University, Taipei, Taiwan; 6 Center for Obesity, Lifestyle and Metabolic Surgery, National Taiwan University Hospital, Taipei, Taiwan

## Abstract

Although many studies have shown that p16^INK4a^ is more highly expressed in the human body during senescence, studies on its relevance to handgrip strength among old adults, are relatively sparse. We enrolled 205 community-dwelling old adults aged 65 years and older without specific medical conditions. Handgrip strength of the dominant hand was measured. Low handgrip strength was defined as the lowest quartile of handgrip strength among the participants. RNA was extracted from peripheral white blood cells. Use quantitative polymerase chain reaction to estimate the p16^INK4a^ mRNA expression level. The average handgrip strength was 25.22 ± 8.98 kg, and gender difference was observed. In the linear regression model, the p16^INK4a^ mRNA expression level was significantly negatively associated with handgrip strength in men but not in women. The β coefficient, representing the change of handgrip strength for each increment in the p16^INK4a^ mRNA expression level, was −0.208 (*p* = 0.024) among old men. The negative association remained after additional covariates adjustment. In the multiple logistic regression model among old men, the odds ratio (OR) of low handgrip strength was 1.246 (*p* = 0.032). In this study, we observed the p16^INK4a^ mRNA expression level was negative associated with handgrip strength among community-dwelling old men.

Low muscle mass with poor physical performance is a crucial problem in geriatric population. A large body of evidence has suggested that poor muscle function could result in increased morbidity, disability, and mortality in long-term follow-up. Handgrip strength is a unique representative performance marker of overall muscular strength used to predict the health outcome in the elderly^1^,^2^,^3^,^4^. Multiple factors that may contribute to the changes of handgrip strength throughout a person’s lifetime have been previously addressed^5^. The possible biological mechanisms linking these factors to reduced grip strength include low-grade inflammation, anabolic energy consumption, and the aging process. However, the molecular markers linking these mechanisms to reduced grip strength are largely unexplored.

In this study, we intended to investigate an ageing-related molecular marker, p16^*INK4a*^ expression in relation to grip strength. The expression of p16^*INK4a*^ has been shown to increase with age in human subjects. p16^*INK4a*^ induces permanent growth arrest termed “cellular senescence.” Association studies have suggested that altered regulation of p16^*INK4a*^ expression may result in human age-associated phenotypes such as type 2 diabetes^6^, atherosclerotic disease^7^, cancer susceptibility^8^,^9^,^10^,^11^, and longevity^12^,^13^,^14^. In mice, delay in some age-related phenotypes such as cataracts and sarcopenia occurs as a result of ablation of p16^*INK4a*^ expressing cells^15^.

Few studies have examined the association between p16^*INK4a*^ expression level and handgrip strength in old persons. This study aims to investigate the connection between aging biomarker and handgrip strength among community-dwelling old adults.

## Methods

### Study population

This is a cross-sectional, observational study. We conduct this study from one medical center (Tri-Service General Hospital, National Defense Medical Center) in northern Taiwan during May, 2012 to April, 2013. People aged 65 years and older who lived in the community of Taipei City eligible for an annual routine geriatric health check-up were the source population. They are functionally independent and relatively healthy in general. All of the participants are Taiwanese older adults. Participants who had cognitive impairment, chest pain and bone pain during exercise, congestive heart failure, regular hemodialysis, or treatment for malignant disease were excluded. At first, trained investigator will screen the subjects by medical record reviewing to find out whether they have the exclusion conditions mentioned above. If they were eligible for this study, the trained investigator will invite them to participate and provide inform consent. The patients’ basic demography, health condition, smoking status, and alcohol consumption and physical activities were reviewed by using a structured questionnaire. Ever smoking in life was defined as a positive smoking status. Alcohol intake was defined as drinking alcohol at least once every week, and was dichotomized. The presence of hypertension was defined according to a self-reported doctor’s diagnosis, the use of antihypertensive medications, or an average blood pressure of ≥140/90 mm Hg. Diabetes mellitus was defined as a self-report of a physician’s diagnosis, a fasting plasma glucose level of ≥126 mg/dL, or the use of diabetic medications (including insulin injection or oral hypoglycemic agents). Medical histories of coronary artery disease, stroke, chronic obstructive pulmonary disease (COPD), or arthritis were ascertained through participants’ self-report. The scores of the brief symptom rating scale (BSRS) and Alzheimer dementia 8 questions (AD-8) were obtained by using a structured questionnaire.

Twenty milliliters of peripheral blood was collected from the participants. After the screening, there were 210 old adults entered our study. There were three participants with incomplete handgrip strength and gait speed measurement (n = 3), two participants with missing data of gait speed test (n = 2). Finally, 205 participants contributed to the final analysis. The participants provided written informed consent before participation. The protocol was approved by the Institutional Review Board of Tri-Service General Hospital, National Defense Medical Center, Taipei, Taiwan. (TSGHIRB 100-05-257). The methods were carried out in accordance with the relevant guidelines and regulations, including any relevant details.

### Functional performance

The handgrip strength of the dominant hand was measured three times with an analogue isometric dynamometer (Exacta Hydraulic Hand Dynamometer; North Coast Medical Inc., Gilroy, CA, USA), and the average value was calculated. Low handgrip strength was defined as the lowest quartile of handgrip strength among the participant. Walking time was measured for all participants over a 15-foot distance as fast as they can. Gait speed was calculated as the distance (m) divided by walking time (sec).

### Measurement of the p16^INK4a^/36B4 mRNA ratio

The total RNA was extracted from peripheral white blood cells with TRIzol reagent (Invitrogen, Carlsbad, CA, USA) and was reverse-transcribed into cDNA. By using Roche Universal Probe library No. 34 (cat. no. 04687671001) as a specific probe, we measured p16^*INK4a*^ with the LightCycler^®^TaqMan^®^Master kit to perform quantitative polymerase chain reaction (qPCR) by using Roche LightCycler (Roche, Mannheim, Germany). The *36B4* gene was used as an internal control. The logarithm of the estimated ratio of p16^*INK4a*^ repeat cycle number to a *36B4* cycle number (p16^*INK4a*^/*36B4* mRNA ratio) [Ct(p16^*INK4a*^ assay)/Ct(*36B4* assay)] indicates the relative expression ratio of p16^*INK4a*^ mRNA. Ct is the fractional cycle number for a threshold fluorescence level to be reached during qPCR. Each assay was performed only once. Using the RNA extracted from Hep3B as the template, the amplification efficiency of p16^*INK4a*^ and *36B4* were respectively 1.89 and 1.92 (close to 2.0)^16^.

### Statistical analysis

The continuous variables of participants’ characteristics are represented as mean ± standard deviation. Categorical variables are expressed as case numbers with percentage. We used multi-variates linear regression to determine the change of handgrip strength for each increment in the p16/*36B4* mRNA ratio. Multi-variates logistic regression analyses between the p16^*INK4a*^/*36B4* mRNA ratio and low handgrip strength were conducted further. By using an extended-model approach for covariate adjustment, three models were investigated: model 1 = age and health behaviors (smoking status and alcohol consumption); model 2 = model 1 plus chronic diseases (hypertension, diabetes mellitus, stroke, coronary artery disease, COPD, arthritis); model 3 = model 2 plus body mass index, white blood cell count, hemoglobin, albumin, BSRS, AD-8 scores and gait speed. All analyses were conducted by using Statistical Package for Social Sciences version 14.0 software (SPSS Inc., Chicago, IL, USA).

## Results

### Characteristics of the study population

The characteristics of the study subjects as a whole are summarized in Table 1. Their mean age was 75.51 ± 7.61 years. Of the participants, 53.17% had hypertension and 13.66% had diabetes mellitus. The average handgrip strength was 25.22 ± 8.98 kg, and sex-specific distribution was observed (*p* < 0.001). Men had higher levels of hemoglobin, and more male participants had COPD and arthritis than the females (*p* = 0.005, 0.026, and 0.006, respectively).

### Associations between the p16 ^INK4a^/36B4 mRNA Ratio and handgrip strength

Initially, we performed a combined gender analysis in the linear regression model. The β coefficient between gender and handgrip strength was 0.754, 0.714, 0.684 from model 1 to model 3 (all *p* < 0.001), which is much stronger than other covariates. We also conduct interaction analysis and it revealed a significant interaction between gender and p16^*INK4a*^/*36B4* mRNA ratio for handgrip strength (*p* = 0.004). Based on these findings, we performed further gender stratified analyses. In the linear model, the p16^*INK4a*^/*36B4* mRNA ratio was significantly associated with handgrip strength in men but not in women in a negative manner (Table 2). After adjusting for age and health behaviors (Model 1), the β coefficient, representing the change of handgrip strength for each increment in the p16^*INK4a*^/*36B4* mRNA ratio, was −0.208 (*p* = 0.024) among old men. After additionally adjusting for other covariates in Models 2 and 3, the β coefficient was −0.232 (*p* = 0.023) and −0.236 (*p* = 0.024), respectively.

We subsequently used a logistic regression model to analyze the associations between p16^*INK4a*^/*36B4* mRNA ratio and low handgrip strength among male participants (Table 3). For each increment in the p16^*INK4a*^/*36B4* mRNA ratio, the odds ratio (OR) for low handgrip strength was 1.211 (95% CI: 1.017–1.444, *p* = 0.032) after controlling for age and health behaviors (Model 1). After controlling other covariates in Models 2 and 3, the association between p16 ^*INK4a*^ mRNA expression level and low handgrip strength remained unchanged; the ORs were 1.230 (95% CI: 1.021–1.482, *p* = 0.030) and 1.246 (95% CI: 1.019–1.524, *p* = 0.032), respectively.

## Discussion

The p16^*INK4a*^ mRNA expression level was indicated as a senescence marker in human subjects. Handgrip strength decreased gradually with aging. This study demonstrated the negative relation between the p16^*INK4a*^ mRNA expression level and handgrip strength among community-dwelling old men. To our best knowledge, this is the first study to examine the association between the p16^*INK4a*^ mRNA expression level and handgrip strength among old adults.

The elevated expression of p16^*INK4a*^ with aging has been reported to limit the regenerative capacity of pancreatic β-cells^17^,^18^ and to alter the repopulating, self-renewal, and homing abilities of hematopoietic stem cells^19^. All those factors declined with age in the mouse forebrain progenitors, and neurogenesis correlated with increased expression of p16^*INK4a*^, which was linked to senescence of neural stem cells^20^. p16^*INK4a*^ induces a permanent growth arrest termed cellular senescence. A common variant of single nucleotide polymorphisms close to the p16^*INK4a*^ genetic region is strongly associated with poor physical function in persons aged 65–80 years^21^. Previous association studies also suggested that altered regulation of p16^*INK4a*^ expression may result in human age-associated phenotypes such as type 2 diabetes^6^, coronary heart disease^7^, cancer susceptibility^8^,^9^,^10^,^11^, and longevity^12^,^13^,^14^. All of these findings support the observation that the increase in p16^*INK4a*^ expression with aging has an impact on human health and leads to morbidity.

The aforementioned studies focus on the relevance between p16^*INK4a*^ expression level and the degenerative tissue or aging organ system; however, studies about the p16^*INK4a*^ mRNA expression level in muscle tissue as well as functional performance in human subjects were relatively sparse. Different methods were used in functional measurements, such as self-reported questionnaires or short physical performance battery scores; however, the results were inconsistent across studies^21^. Handgrip strength is a representative marker of overall functional performance; a higher p16^*INK4a*^ mRNA expression level implies that aging is occurring in the human body. The negative correlation between the p16^*INK4a*^ mRNA expression level and handgrip strength in our study suggested that the muscle tissue of the upper arm, which is responsible for handgrip strength, degenerates and produces worse performance during the process of aging. This is the first study to explore the relation between the p16^*INK4a*^ mRNA expression level and handgrip strength in the elderly population. The possible mechanism of p16^*INK4a*^ expression resulting in muscle loss and subsequently leading to functional decline merits further investigation.

A number of factors are known to be implicated with muscle strength decline, including a sedentary lifestyle^5^, excess body weight^22^, smoking^23^, cardiovascular disease, hypertension^5^, and diabetes mellitus^5^,^24^. Anemia is associated with lower handgrip strength in community-dwelling older persons even after adjustment for inflammatory markers such as C-reactive protein, interleukin-6, and tumor necrosis factor-α^25^. In this study, we examined the relation between the p16^*INK4a*^ mRNA expression level, an aging biomarker, and handgrip strength with adjustment for multiple covariates that had been reported in previous studies. We observed that the p16^*INK4a*^ mRNA expression level was negatively associated with handgrip strength in old men. This negative relation also existed among old women; however, it did not reach statistical significance.

The discrepancy in statistical significance on the negative association between the p16^*INK4a*^ mRNA expression level and handgrip strength in different sex groups might be because the mean age of men was higher than that of women, resulting in a relatively low level of p16^*INK4a*^ mRNA expression in the female participants. Second, comorbidities such as diabetes, obesity, stroke, and COPD were much more prevalent in men than in women, and all of these illnesses accelerate the aging process, leading to an increase in the p16^*INK4a*^ mRNA expression level. In addition, previous studies disclosed that handgrip strength is additionally influenced by body mass index in men but not in women^26^. In our study, men had a higher body mass index; moreover, cigarette smoking was more prevalent among men, leading to the simultaneous increase in the p16^*INK4a*^ expression level and decrease in handgrip strength.

Our findings have some clinical applications. First, a higher p16^*INK4a*^ mRNA expression level was associated with lower handgrip strength. This result indicates the potential role of the p16^*INK4a*^ mRNA expression level of peripheral white blood cells on poor physical performance estimation, especially the upper limbs. Second, handgrip strength is an easy-to-measure indicator of physical function in clinical practice; regular measurement and comparison to detect aging process related functional decline is important. Moreover, for each increment in the p16^*INK4a*^ mRNA expression, the odds of low handgrip strength increased 24.6% in men after controlling potential covariates. Although p16^*INK4a*^ mRNA was obtained from peripheral white blood cells in our study, this association in men may provide a clue for clinical and basic mechanistic research.

There are several limitations in our study. First, this is a cross-sectional study that enrolled only a few participants at only one medical center; moreover, a causality between p16^*INK4a*^ mRNA expression level and handgrip strength could not be established. Second, our study enroll old adults aged 65 years and older who live in the community are relative healthy and ambulatory. Although we try to adjust multiple covariates in the regression model to minimize the possible confounding effects, this observation may not be applied to the elderly population with severe illness leading to the limited generalizability to other population. Third, the estimated power for two-sample comparison of means of p16^*INK4a*^/*36B4* mRNA ratio between male group with and without low handgrip strength was 62%. However, even though our study had a few sample size and unsatisfied statistical power, the p16^*INK4a*^/*36B4* mRNA ratio was significantly associated with handgrip strength in men. In addition, the p16^*INK4a*^ mRNA expression level was measured from peripheral white blood cells, not from muscle cells. Also, we did not estimate the participants’ muscle mass; thus, the distribution of p16^*INK4a*^ mRNA expression level in different muscle types could not be analyzed in this study. Furthermore, few inflammatory biomarkers were measured in this study; however, the relevant comorbidities mentioned in previous studies were included.

## Conclusion

In this study, we found that the p16^*INK4a*^ mRNA expression level is negatively associated with handgrip strength among community-dwelling old men. Further investigations of the causality between the p16^*INK4a*^ mRNA expression level of peripheral white blood cells and muscle mass, as well as physical performance, are necessary.

## Additional Information

**How to cite this article**: Kao, T.-W. *et al*. Examining how p16^*INK4a*^ expression levels are linked to handgrip strength in the elderly. *Sci. Rep.* **6**, 31905; doi: 10.1038/srep31905 (2016).

## Figures and Tables

**Table 1:** Characteristics of the study participants.

Characteristics	Men (n = 98)	Women (n = 107)	Total (N = 205)	*p* value
Continuous variables				
Age, years, mean (SD)	78.34 (8.30)	72.92 (5.85)	75.51 (7.61)	<0.001
BMI, kg/m^2^, mean (SD)	24.35 (2.68)	24.06 (3.23)	24.19 (2.98)	0.062
Handgrip strength, kg, mean (SD)	31.45 (8.05)	19.51 (5.21)	25.22 (8.98)	<0.001
Gait speed, m/sec, mean (SD)	0.87 (0.25)	0.91 (0.24)	0.89 (0.25)	0.598
WBC count, 10^3^/μL, mean (SD)	5.51 (1.52)	5.29 (1.37)	5.39 (1.44)	0.132
Hemoglobin, g/dL, mean (SD)	14.09 (1.36)	13.20 (1.00)	13.62 (1.26)	0.005
Albumin, mg/dL, mean (SD)	4.43 (0.24)	4.48 (0.20)	4.46 (0.22)	0.094
BSRS, mean (SD)	1.87 (2.77)	2.53 (2.79)	2.22 (2.79)	0.850
AD-8 score, mean (SD)	0.63 (1.02)	0.90 (1.29)	0.78 (1.17)	0.836
p16^*INK4a*^/*36B4* mRNA ratio, mean (SD)	0.86 (2.06)	0.70 (1.30)	0.78 (1.70)	0.350
Categorical variables				
Smoking, n (%)	25 (25.51)	3 (2.80)	28 (13.66)	<0.001
Alcohol consumption ≥once weekly, n (%)	11 (11.22)	0 (0)	11 (5.36)	<0.001
Hypertension, n (%)	52 (53.06)	57 (53.27)	109 (53.17)	0.955
Diabetes mellitus, n (%)	15 (15.31)	13 (12.15)	28 (13.66)	0.488
Stroke, n (%)	5 (5.10)	4 (3.74)	9 (4.39)	0.632
Coronary artery disease, n (%)	10 (10.20)	11 (10.28)	21 (10.24)	0.968
COPD, n (%)	17 (17.35)	8 (7.48)	25 (12.19)	0.026
Arthritis, n (%)	26 (26.53)	47 (43.93)	73 (35.61)	0.006

Abbreviations:AD-8, Alzheimer dementia 8 questions; BMI, body mass index; BSRS, brief symptom rating scale; COPD, chronic obstructive pulmonary disease; SD, standard deviation; WBC, white blood cells.

**Table 2:** Regression coefficients for the association between p16^*INK4*^ a/*36B4* mRNA ratio and handgrip strength.

Models^a^	Men		Women	
	*β*^b^ (S.E.)	*p* value	*β*^b^ (S.E)	*p* value
Model 1	−0.208 (0.119)	0.024	−0.076 (0.107)	0.448
Model 2	−0.232 (0.132)	0.023	−0.054 (0.105)	0.585
Model 3	−0.236 (0.135)	0.024	−0.080 (0.097)	0.380

^a^Adjusted covariates: model 1 = age and health behaviors, model 2 = model 1 + chronic diseases, model 3 = model 2 + body mass index, WBC counts, hemoglobin, albumin, BSRS, AD-8 scores, and gait speed.

^b^*β* Coefficient was interpreted as the change of handgrip strength for each one increment in the p16^*INK4a*^/*36B4* mRNA ratio. Abbreviations: AD-8,Alzheimer dementia 8 questions; LDL, low-density lipoprotein; S.E., standard error; WBC, white blood cells.

**Table 3:** Logistic regression for the association between the p16^*INK4*^ a/*36B4* mRNA ratio and low handgrip strength in men.

Models^a^	OR^b^ (95% CI)	*p* value
Model 1	1.211 (1.017–1.444)	0.032
Model 2	1.230 (1.021–1.482)	0.030
Model 3	1.246 (1.019–1.524)	0.032

^a^Adjusted covariates: model 1 = age and health behaviors, model 2 = model 1 + chronic diseases, model 3 = model 2 + body mass index, WBC counts, hemoglobin, albumin, BSRS, AD-8 scores, and gait speed.

^b^OR of low handgrip strength for each increment in the p16^*INK4a*^/*36B4* mRNA ratio. Abbreviations: AD-8, Alzheimer dementia 8 questions; BSRS, brief symptom rating scale; CI, confidence interval; OR, odds ratio; WBC, white blood cells.
